# 

**Published:** 1862-08

**Authors:** 


New Series, Vol. 5, No. 8. Whole Series, Vol. 19, No. 8.

Dr. R. C. Hamill
Chicago
Box 1305

### THE CHICAGO

### MEDICAL JOURNAL.

DANIEL BRAINARD, M. D.,
J. ADAMS ALLEN, M. D.,
Editors and Proprietors.

AUGUST, 1862.

CHICAGO:

WM. PIGOTT, BOOK AND JOB PRINTER, 130 CLARK STREET.

1862.

